# RELATIONSHIPS BETWEEN LEISURE CONSTRAINTS AND MODES OF TRANSPORTATION AMONG OLDER ADULTS IN SUBSIDIZED HOUSING

**DOI:** 10.1093/geroni/igac059.802

**Published:** 2022-12-20

**Authors:** Junyan Tian, Shyuan Ching Tan, Angie Sardina, Alyssa Gamaldo, Lesley Ross

**Affiliations:** Penn State University, State College, Pennsylvania, United States; California State University San Marcos, San Marcos, California, United States; UNC Wilmington, Wilmington, North Carolina, United States; Pennsylvania State University, State College, Pennsylvania, United States; Clemson University, Clemson, South Carolina, United States

## Abstract

Transportation needs among lower-income older adults is understudied, particularly regarding how transportation utilization is related to leisure constraints. This preliminary study included 39 adults residing in subsidized housing in North Carolina and Pennsylvania (M=68.03, SD=10.26, female=74.4%) and assessed reported modes of transportation for daily activities and perceived leisure activity constraints. Much of the sample reported driving (57.9%) or relying on others to drive (70.3%) with a significant sample reporting use of public transportation (48.6%). Transportation utilization was differentially correlated with leisure constraints. Perceived difficulties getting to/from activities in the community was associated with greater utilization of having ‘others drive you’ (r=0.43, p=.011), but lower utilization of ‘driving oneself’ (r=-0.40, p=.019). Walking as a mode of transportation was associated with lower (r=-0.41, p=.014) perceived difficulties getting to/from activities in the housing complex. These results indicate the importance of further exploring the association between transportation and leisure needs of these lower-income older adults.

